# Präklinisches Loading bei Patienten mit akutem Thoraxschmerz und akutem Koronarsyndrom – PRELOAD-Umfrage

**DOI:** 10.1007/s00063-023-01087-8

**Published:** 2023-11-30

**Authors:** Sascha Macherey-Meyer, Simon Braumann, Sebastian Heyne, Max Maria Meertens, Tobias Tichelbäcker, Stephan Baldus, Samuel Lee, Christoph Adler

**Affiliations:** Medizinische Fakultät und Uniklinik Köln, Klinik III für Innere Medizin, Universität zu Köln, Kerpener Str. 62, 50937 Köln, Deutschland

**Keywords:** Antikoagulanzien, Thrombozytenaggregationshemmung, Myokardinfarkt, STEMI, NOAK, Heparin, Anticoagulants, Antiplatelets, Myocardial infarction, STEMI, NOAC, Heparin

## Abstract

**Hintergrund:**

Leitlinien zum Myokardinfarkt (MI) empfehlen eine blutverdünnende Therapie zum Diagnosezeitpunkt. Während der MI mit ST-Streckenhebung (STEMI) präklinisch sicher detektiert werden kann, ist das akute Koronarsyndrom ohne ST-Streckenhebung (NSTE-ACS) eine Arbeitsdiagnose.

**Zielsetzung:**

Erfassung von präklinischem Loading mit Acetylsalicylsäure (ASS) und Heparin stratifiziert nach ACS-Entität und in Abhängigkeit von oraler Antikoagulation.

**Methoden:**

Die PRELOAD-Studie wurde als deutschlandweite Online-Umfrage durchgeführt. STEMI/NSTE-ACS-Szenarien wurden mit folgender Variation präsentiert: I) ohne Vorbehandlung, II) Vorbehandlung mit neuem oralem Antikoagulan (NOAK), Vorbehandlung mit Vitamin-K-Antagonist (VKA). Loading-Strategien wurden erhoben und umfassten: a) ASS, b) unfraktioniertes Heparin (UFH), c) ASS + UFH, d) kein Loading.

**Ergebnisse:**

In die Studie wurden 708 Notfallmediziner*innen eingeschlossen. Beim NSTE-ACS ohne Vorbehandlung entschieden sich 79 % für ein Loading (*p* < 0,001). ASS + UFH (71,4 %) war die häufigste Antwort. Beim STEMI entschlossen sich 100 % zum Loading, wobei 98,6 % ASS + UFH wählten. Beim NSTE-ACS mit NOAK-Vorbehandlung wählten 69,8 % Loading (*p* < 0,001). Eine VKA-Einnahme führte in 72,3 % der Fälle zum Loading (*p* < 0,001). ASS gefolgt von ASS + UFH waren die häufigsten Antworten. Beim STEMI war eine NOAK- bzw. VKA-Behandlung in 97,5 bzw. 96,8 % der Fälle mit einer Loading-Entscheidung verbunden (*p* < 0,001) – vermehrt wurde eine ASS-Monotherapie eingesetzt.

**Schlussfolgerungen:**

Präklinisches Loading ist die präferierte Behandlungsstrategie, obwohl beim NSTE-ACS die Leitlinien eine Antikoagulation erst zum Zeitpunkt der Diagnose empfehlen. Im Fall vorbestehender oraler Antikoagulation wird präklinisches Loading gehäuft in Form einer ASS-Monotherapie durchgeführt. Beim STEMI mit Notwendigkeit der sofortigen invasiven Strategie bedeutet dies eine potenzielle Unterversorgung.

**Zusatzmaterial online:**

Zusätzliche Informationen sind in der Online-Version dieses Artikels (10.1007/s00063-023-01087-8) enthalten.

## Abstract (en)

**Background:**

Guidelines on myocardial infarction (MI) recommend antithrombotic and anticoagulatory treatment at time of diagnosis. MI with ST segment elevation (STEMI) is mostly a certain diagnosis. Acute coronary syndrome without ST segment elevation (NSTE-ACS) has diagnostic uncertainty and remains a working diagnosis in the prehospital setting.

**Objective:**

Assessment of prehospital loading with aspirin and heparin depending on ACS subtype and pretreatment with oral anticoagulants.

**Methods:**

The PRELOAD survey was a nationwide German study. STEMI/NSTE-ACS scenarios were designed and varied in pretreatment: I) no pretreatment, II) new oral anticoagulants (NOAC), III) vitamin K antagonist (VKA). Loading strategy was assessed and included: a) aspirin (ASA), b) unfractionated heparin (UFH), c) ASA + UFH, d) no loading.

**Results:**

A total of 708 emergency physicians were included. In NSTE-ACS without pretreatment, 79% chose loading (*p* < 0.001). ASA + UFH (71.4%) was the preferred option. In corresponding STEMI scenario, 100% chose loading and 98.6% preferred ASA + UFH (*p* < 0.001). In NSTE-ACS with NOAC pretreatment, 69.8% favored loading (*p* < 0.001); in VKA pretreatment the corresponding rate was 72.3% (*p* < 0.001). In each scenario, ASA was the preferred option. In STEMI with NOAC pretreatment, 97.5% chose loading (*p* < 0.001); analogous rate was 96.8% in STEMI with VKA pretreatment (*p* < 0.001). ASA was the preferred option again.

**Conclusions:**

Prehospital loading was the preferred treatment strategy despite the diagnostic uncertainty in NSTE-ACS and guidelines recommending loading at time of diagnosis. Pretreatment with oral anticoagulants resulted in a strategy shift to loading with only aspirin. In STEMI patients, this indicates potential undertreatment.

Akuter Thoraxschmerz ist ein häufiger Alarmierungsgrund im Rettungsdienst und hat verschiedene Ursachen [1, 2]. Eine wichtige Differenzialdiagnose ist der Myokardinfarkt (MI), der Behandlungspfad folgt häufig diesem bedrohlichen Krankheitsbild [3, 4]. Während ein MI mit ST-Streckenhebung (STEMI) mit hoher diagnostischer Sicherheit prähospital detektiert werden kann [5, 6], ist das akute Koronarsyndrom ohne ST-Streckenhebung (NSTE-ACS) eine Arbeitsdiagnose. NSTE-ACS definiert sich über eine instabile Angina pectoris oder einen Myokardzellschaden mit klinischen Zeichen der Ischämie [7]. Die Bestimmung von Troponin ist relevant, präklinisch technisch möglich, aber erfolgt in Deutschland nicht routinemäßig [8, 9]. Prähospital besteht beim NSTE-ACS eine erhebliche diagnostische Ungenauigkeit [10].

Europäische Leitlinien zur Behandlung des MI empfehlen die Gabe von Acetylsalicylsäure und einem parenteralen Antikoagulans unmittelbar *zum Diagnosezeitpunkt* [3, 5, 11]. In Deutschland erfolgt diese Vorbehandlung, die im klinischen Alltag auch als „Loading“ zusammengefasst wird, häufig durch Notärzt*innen (NÄ) vor Ankunft im Krankenhaus [10, 12]. Diese Praxis birgt potenziellen Schaden bei Diagnosefehlern (Aortensyndrom, Pneumothorax) oder unerwünschten Nebenwirkungen (Blutungen, Blutbildveränderungen) in sich [3, 4]. Das Spannungsfeld aus eingeschränkter diagnostischer Sicherheit, Unterversorgung und potenziellem Schaden stellt eine tägliche Herausforderung beim NSTE-ACS dar. Die Folge können heterogene Herangehensweisen sein, und das Loading-Dilemma wird durch eine vorbestehende orale Antikoagulation mittels neuer oraler Antikoagulanzien (NOAK) oder Vitamin-K-Antagonisten (VKA) verschärft.

## Methoden

### Studiendesign und Rationale

Die PRELOAD-Studie wurde vom 06.11. bis 20.11.2022 als szenarienbasierte Umfrage in Deutschland mit den Zielen durchgeführt,

1. die Loading-Entscheidung bei Patienten mit akutem Thoraxschmerz und ACS durch Befragung von NÄ zu erfassen;
2. das Verhalten von NÄ bei vorbestehender oraler Antikoagulation zu erfassen.

In der Definition des ACS wurde NSTE-ACS vom STEMI abgegrenzt. Es wurde je ein Szenario ohne medikamentöse Vorbehandlung, eines mit NOAK- und eines mit VKA-Vorbehandlung erstellt (s. Zusatzmaterial Fragebogen).

Zusätzlich wurde der Fall mit kardiogenem Schock (NSTE-ACS) und Indikation zur sofortigen invasiven Koronardiagnostik ergänzt. Kriterien für die sofortige invasive Therapie sind im Anhang beigefügt (Zusatzmaterial Tab. 1, nach [13–15]).

Die Diagnose STEMI/NSTE-ACS wurde explizit vorgegeben. Folgende Antwortmöglichkeiten standen zur Verfügung:

a) Acetylsalicylsäure (ASS), b) unfraktioniertes Heparin (UFH), c) ASS + UFH und d) kein Loading.

Zusätzlich wurde gefragt, ob nach Ansicht der Befragten explizite Leitlinienempfehlungen zum präklinischen Loading beim STEMI und beim Myokardinfarkt ohne ST-Streckenhebung (NSTEMI) bestehen. Neben Ja und Nein konnte hier auch gewählt werden, dass die Leitlinien nicht ausreichend bekannt seien.

Die Studie wurde im Einklang mit der Deklaration von Helsinki durchgeführt und durch die Ethikkommission der Universität zu Köln zustimmend bewertet (Nr. 22-1400, 11/2022).

### Teilnehmende

Die Studienteilnehmer*innen wurden über geschlossene Informationskanäle der Rettungsdienstsysteme (bspw. Nachrichtenmessenger-Gruppen) oder über einen Social-Media-Nachrichtendienst (Twitter) über die Umfrage informiert. Hierüber wurde der Teilnahme-Link bereitgestellt. Die Studieninformation beschrieb die Tätigkeit als NÄ als Teilnahmevoraussetzung. Die freiwillige Studienteilnahme war mit der Einwilligung in die Datenschutzerklärung verbunden. Die Teilnehmenden gaben ausschließlich anonymisierte Daten an. Für die Auswertung wurden nur vollständige Antwortbögen berücksichtigt. Pro IP-Adresse konnte nur eine Teilnahme erfolgen.

### Statistische Analyse

Die statistische Auswertung erfolgte als deskriptive Statistik durch Ermittlung des Medians und des Mittelwerts für metrische Variablen oder Darstellung der Häufigkeiten bzw. der Verteilung von kategorialen Variablen. Die analytische Statistik wurde für die abhängige Zielvariable „Loading-Entscheidung“ mittels T‑Test und Chi-Quadrat-Test durchgeführt. Zusätzlich wurden Variablen (Alter, Geschlecht, Diensterfahrung, Einsatzzeiten, Einsatzgebiet, Fachgebiet) in einer binären logistischen Regressionsanalyse hinsichtlich des Einflusses auf die abhängige Variable „Loading-Entscheidung“ untersucht. Das zweiseitige Signifikanzniveau alpha wurde je als < 0,05 definiert. Die Analysen erfolgten mittels IBM SPSS Statistics (Fa. IBM Corp., Armonk, NY, USA).

## Ergebnisse

### Merkmale der Studienpopulation

Zum Studienabschluss wurden von 1205 Proband*innen Antwortbögen zurückgesendet. Von diesen übermittelten 708 (58,8 %) einen vollständigen Antwortbogen. Diese Kohorte stellte gemäß Protokoll die Stichprobe dar. Das mittlere Alter der Proband*innen war 39,7 (± 8,5) Jahre, die mittlere Diensterfahrung in der Notfallmedizin betrug 7,9 (± 10,2) Jahre (Tab. 1). Insgesamt waren 490 (69,2 %) männlich und 411 (58,1 %) waren im Fachgebiet Anästhesie tätig. Es arbeiteten 496 (70,1 %) ≤ 72 h pro Monat in der Notfallmedizin. Die überwiegenden Einsatzgebiete waren urban geprägt.

**Tab. 1:** Baseline-Charakteristiken der Studienkohorte

	708 Proband*innen (%), [Standardabweichung]		*p*-Wert
*Alter*	Mittelwert	39,7 [± 8,5]	< 0,001
*Geschlecht*	Männlich	490 (69,2)	–
	Weiblich	216 (30,5)	
	Divers	2 (0,3)	
*Dienstjahre Notfallmedizin*	Mittelwert	7,9 [± 10,2]	< 0,001
*Einsatzzeiten Notfallmedizin*	< 24 h	101 (14,3)	–
	24–72 h	395 (55,8)	
	72–120 h	140 (19,8)	
	> 120 h	72 (10,2)	
*Einsatzgebiet Notfallmedizin*	Metropole	109 (15,4)	–
	Großstadt	230 (32,5)	
	Mittelstadt	221 (31,2)	
	Ländliche Region	148 (20,9)	
*Fachgebiet*	Anästhesie	411 (58,1)	–
	Chirurgie	55 (7,8)	
	Innere Medizin und Kardiologie	66 (9,3)	
	Innere Medizin ohne Kardiologie	117 (16,5)	
	Andere Fachrichtung	59 (8,3)	

### Loading beim ACS ohne Vorbehandlung

**Abb. 1:**
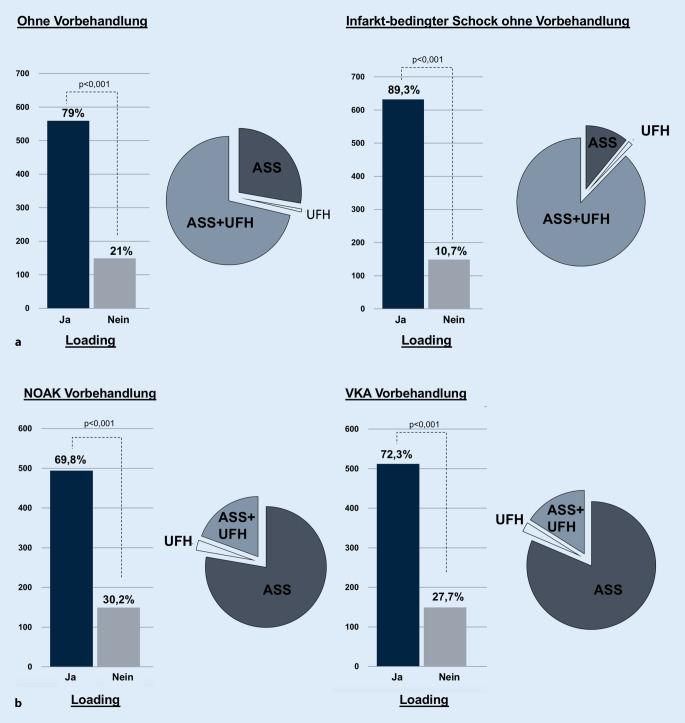
**a** Loading-Entscheidung beim NSTE-ACS ohne Vorbehandlung. **b** Loading-Entscheidung beim NSTE-ACS mit oraler Antikoagulation. *ASS* Acetylsalicylsäure, *UFH* unfraktioniertes Heparin, *NOAK* neues orales Antikoagulans, *VKA* Vitamin-K-Antagonist

Beim NSTE-ACS-Szenario ohne Vorbehandlung entschieden sich 79 % für ein Loading (*p* < 0,001; Tab. 2a). In der Loading-Subgruppe waren ASS + UFH (71,4 %) gefolgt von ASS (27,7 %) die häufigsten Antworten (Abb. 1a).

**Tab. 2:** Loading-Entscheidung bei akutem Koronarsyndrom

	Loading (%)	Kein Loading (%)	*p*-Wert*
*a: NSTE-ACS*			
NSTE-ACS ohne Vorbehandlung	559 (79)	149 (21)	< 0,001
NSTE-ACS mit NOAK-Vorbehandlung	494 (69,8)	214 (30,2)	< 0,001
NSTE-ACS mit Phenprocoumon-Vorbehandlung	512 (72,3)	196 (27,7)	< 0,001
NSTE-ACS mit kardiogenem Schock	632 (89,3)	76 (10,7)	< 0,001
*b: STEMI*			
STEMI ohne Vorbehandlung	708 (100)	0 (0)	–
STEMI mit NOAK-Vorbehandlung	690 (97,5)	18 (2,5)	< 0,001
STEMI mit Phenprocoumon-Vorbehandlung	685 (96,8)	23 (3,2)	< 0,001

*NSTE-ACS* akutes Koronarsyndrom ohne ST-Streckenhebung, *STEMI* akutes Koronarsyndrom mit ST-Streckenhebung, *NOAK* neues orales Antikoagulans, – nicht bestimmbar

*t-Test einer Stichprobe

Das NSTE-ACS-Szenario mit kardiogenem Schock wurde von 89,3 % mit einem Loading beantwortet (*p* < 0,001; Tab. 2a). In 87,8 % der Fälle wurde die Kombination ASS + UFH gewählt (Abb. 1a).

**Abb. 2:**
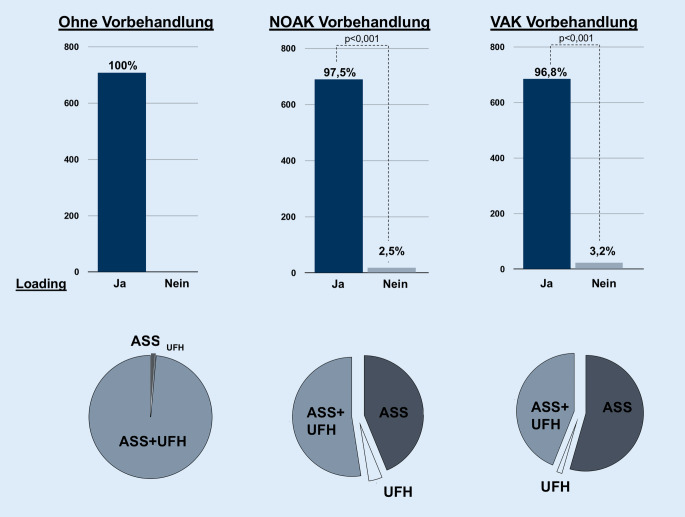
Loading-Entscheidung beim STEMI. *ASS* Acetylsalicylsäure, *UFH* unfraktioniertes Heparin, *NOAK* neues orales Antikoagulans, *VKA* Vitamin-K-Antagonist

Beim STEMI entschlossen sich 100 % zum Loading, wobei 98,6 % die Kombination aus ASS + UFH wählten (Tab. 2b und Abb. 2).

### Loading bei NSTE-ACS mit vorbestehender Antikoagulation

Das NSTE-ACS-Szenario mit NOAK-Vorbehandlung resultierte in 69,8 % in Loading (*p* < 0,001; Tab. 2a). In der Loading-Subgruppe wurde ASS (77,7 %) gefolgt von ASS + UFH (19,4 %) am häufigsten gewählt (Abb. 1). Die Vorbehandlung mit VKA führte in 72,3 % zum Loading (*p* < 0,001; Tab. 2a). In der Loading-Subgruppe waren ASS (81,4 %) gefolgt von ASS + UFH (16 %) die häufigsten Antworten (Abb. 1b).

### Loading bei STEMI mit vorbestehender Antikoagulation

Das STEMI-Szenario mit NOAK-Vorbehandlung resultierte in 97,5 % in einem Loading (*p* < 0,001; Tab. 2b). In der Loading-Subgruppe wurde ASS + UFH (52,5 %) gefolgt von ASS (43,8 %) am häufigsten gewählt (Abb. 2). Die Vorbehandlung mit einem VKA führte in 96,8 % zum Loading (*p* < 0,001; Tab. 2b). In der Loading-Subgruppe waren ASS (54,5 %) gefolgt von ASS + UFH (43,9 %) die häufigsten Antworten (Abb. 2).

In der Einschätzung der Leitlinienempfehlungen zum präklinischen Loading konnten folgende Ergebnisse beobachtet werden: Beim STEMI gaben 19,5 % an, dass keine Leitlinienempfehlungen bestünden, 16,9 % gaben an, die gültigen Leitlinienempfehlungen nicht ausreichend zu kennen, um die Frage zu beantworten. Weiterhin gaben 63,6 % an, dass explizite Leitlinienempfehlungen existierten. Korrespondierende Raten für NSTEMI waren 44,4, 18,5 und 37,1 %.

### Regressionsanalyse

Das NSTE-ACS-Szenario ohne Vorbehandlung wurde mit steigendem Alter (OR: 1,02; 95%-KI: 1,001–1,047), steigender Diensterfahrung (OR: 1,029; 95%-KI: 1,003–1,056) und bei längeren monatlichen Einsatzzeiten (OR: 2,63; 95%-KI : 1,11–6,23) häufiger mit einem Loading beantwortet (vgl. Zusatzmaterial Tab. 2a).

Sowohl im NSTE-ACS-NOAK- als auch im NSTE-ACS-VKA-Szenario war das männliche Geschlecht mit einer höheren Wahrscheinlichkeit für eine Loading-Entscheidung verbunden (OR: 1,66; 95%-KI: 1,18–2,33 und OR: 1,67; 95%-KI: 1,18–2,36).

Geringeres Alter war beim STEMI-Szenario mit VKA mit einer höheren Wahrscheinlichkeit für Loading verbunden (OR: 0,95; 95%-KI: 0,91–0,99). Die übrigen Einflussfaktoren zeigten szenarienübergreifend keinen signifikanten Effekt (vgl. Zusatzmaterial Tab. 2b).

## Diskussion

Es konnten 1,5 % aller berufstätigen deutschen NÄ erreicht werden [16]. Es handelt sich um die größte überregionale Befragung zum Loading bei akutem Thoraxschmerz und ACS. Die Umfrage zeigt, dass:

- präklinisches Loading mit ASS + UFH beim ACS die bevorzugte Strategie ist;
- eine vorbestehende orale Antikoagulation mehrheitlich zu einem Strategiewechsel mit einer ASS-Monotherapie führt;
- bei STEMI-Patienten mit oraler Antikoagulation ein Versorgungsdefizit durch die bevorzugte ASS-Monotherapie möglich ist.

Präklinisches Loading war in der Befragung die häufigste Behandlungsstrategie beim ACS. Dies deckt sich mit vorangegangenen Untersuchungen [10, 12]. Zum gegenwärtigen Zeitpunkt gibt es auf deutscher und europäischer Ebene Leitlinienempfehlungen und Positionspapiere zur antithrombozytären und antikoagulatorischen Behandlung des ACS. Die Europäische und Deutsche Gesellschaft für Kardiologie (ESC, DGK) empfehlen eine parenterale Antikoagulation als Zusatz zur Plättchenhemmung *zum Zeitpunkt der Diagnose* und im Fall des NSTE-ACS insbesondere während Revaskularisierungsverfahren [3, 5, 11, 14]. Die Umfrage zeigte beim Loading einen konstanten ASS-Einsatz – obwohl hier eine Evidenzlücke besteht und die Effektivitätsprüfung für die präinterventionelle Behandlungsphase bisher nicht in randomisierten Studien gezeigt werden konnte. Es zeigte sich in der Umfrage eine entscheidende Varianz in der Heparintherapie, sodass hier der Fokus der Betrachtung liegen soll.

### Präinterventionelle Heparintherapie beim STEMI

In der STEMI-Behandlung konnte eine prähospitale Heparinbehandlung bis dato zwar die Rate verschlossener Koronargefäße senken, jedoch keinen signifikanten Einfluss auf kardiovaskuläre Major-Endpunkte oder die 30-Tages-Mortalität zeigen [17–19]. In der kürzlich erschienenen ESC-Leitlinie zur Behandlung des ACS wurde die Evidenzlücke in der Heparintherapie während der präinterventionellen Behandlungsphase erstmalig in den Fokus gerückt, der Einsatz wird jedoch bis auf Weiteres zum Zeitpunkt der Diagnose empfohlen [11]. Loading wurde im Einklang mit diesen Leitlinienempfehlungen auch in der Befragung bei den STEMI-Szenarien durchgeführt. Lediglich 2/3 der NÄ gab an, die Leitlinienempfehlungen zu kennen. Bemerkenswert ist die Tendenz zur ASS-Monotherapie bei oral antikoagulierten Patienten. Die Leitlinie erwähnt diese Patientengruppe nicht explizit. Stattdessen gibt es für den klinischen Alltag als Orientierung ein Positionspapier der DGK zum Loading bei Patienten mit *akutem Koronarsyndrom unter NOAK-Therapie* [20]*. *Unter NOAK-Vorbehandlung sollen beim STEMI ASS und UFH immer appliziert werden. Für VKA fehlt es an expliziten Empfehlungen. Die Abkehr von der Heparinvorbehandlung in der Umfrage ist somit diskutabel und Bedarf einer weiteren Aufarbeitung in Studien. Die Umfrage zeigt hier auch eine mögliche Unsicherheit auf Seiten der NÄ auf.

### Präinterventionelle Heparintherapie beim NSTE-ACS

Bei notärztlich vorgestellten Patienten mit akutem Thoraxschmerz wird präklinisches Loading in Real-World-Daten häufig praktiziert, jedoch hat nur ein Bruchteil ein NSTE-ACS [10]. Im Detail werden 40 % der Patienten gemessen an der Entlassdiagnose ohne Indikation mit Blutverdünnern behandelt [10]. Es besteht beim NSTE-ACS auf Basis von dieser Voruntersuchung mit präklinischer diagnostischer Unsicherheit offensichtlich eine Übertherapie mittels Loading, deren begründende Motive letztlich zu prüfen sind. Das vollständige Ausmaß ist durch fehlende systematische Erfassung möglicherweise unterschätzt.

NSTE-ACS umfasst eine heterogene Patientengruppe mit instabiler Angina pectoris. Dies kann bis zur hämodynamischen Instabilität und zum Kreislaufstillstand reichen – so variabel wie die Kohorte ist das Patientenmanagement. Dieses erstreckt sich von sofortiger invasiver Diagnostik über selektive Koronarangiographie bis hin zur Ambulantisierung der Anschlussdiagnostik [3, 11]. Dennoch empfiehlt hier die ESC-Leitlinie ebenfalls die kombinierte Plättchenhemmung und Antikoagulation zum Zeitpunkt der Diagnose. Das European Resuscitation Council folgt inhaltlich dieser Empfehlung, weist jedoch darauf hin, dass es keine wissenschaftlichen Hinweise für eine Überlegenheit der prä- im Vergleich zur innerklinischen Behandlung gebe [4]. Im Positionspapier der DGK zum Loading bei Patienten mit *akutem Koronarsyndrom unter NOAK-Therapie* kommt die Autorengruppe zur Einschätzung, dass beim NSTE-ACS hingegen die Heparintherapie von der Abwägung des Blutungs- und Ischämierisikos abhängig zu machen ist [20]. Belastbare Befunde liegen hierzu präklinisch zumeist nicht vor, es besteht ein Informationsdefizit. Sollte es Zweifel an der NOAK-Einnahme geben, sei eine Heparintherapie aber indiziert [20]. Ein Positionspapier der Acute Cardiovascular Care Association der ESC resümiert, dass der Nutzen einer prähospitalen ASS- oder Heparintherapie beim NSTE-ACS gänzlich ungeklärt sei [21]. Eine präklinische Heparintherapie wird hier nur im Fall einer sofortigen invasiven Strategie (Koronardiagnostik innerhalb von 2 h) empfohlen [21]. Die europäische Empfehlung fällt somit bei gleicher Studienlage deutlich defensiver aus. In diesem Spannungsfeld aus konkurrierenden Empfehlungen bewegen sich NÄ beim NSTE-ACS täglich. Diskutabel sind im Besonderen die Empfehlungen der ESC/DGK-Leitlinien bezüglich der Antikoagulationstherapie *zum Zeitpunkt der Diagnose.* Diese begründen sich auf zwei Metaanalysen aus den Jahren 1996 und 2000 [22, 23]. Die additive *innerklinische, kontinuierliche *UFH-Gabe zeigte in der gepoolten Analyse (*n* = 1353 Patienten) aus 6 Studien eine Reduktion um 33 % für das Risiko des kombinierten Endpunktes Reinfarkt und Mortalität (OR: 0,67; 95%-KI: 0,45–0,99) [23]. Die Heparintherapie hatte keinen Einfluss auf die Häufigkeit von Angina pectoris, die Revaskularisations- oder Blutungsrate [23]. Die Studien hatten relevante Störfaktoren. Es wurden STEMI-Patienten berücksichtigt [24, 25], die Quote für Linksherzkatheter war variabel (20–91 %), und die Revaskularisationsrate war gemessen an heutigen Kennzahlen niedrig (1,8–50 %) [22, 24–29]. Patienten mit vorbestehender Antikoagulationstherapie wurden ausgeschlossen [24–29]. Diese Studienergebnisse, die heute herangezogen werden, um die Heparintherapie *zum Zeitpunkt der Diagnose *begründen, sind selbst nicht unmittelbar auf das heutige Patientenkollektiv anwendbar. Sie lassen keinerlei Rückschlüsse auf eine *präklinische* Antikoagulation zu. Das gegenwärtige ACS-Management umfasst zusätzlich mit differenzierten Interventionsstrategien, neuen Stentgenerationen sowie potenteren Thrombozytenaggregationshemmern wichtige Einflussfaktoren für die blutverdünnende Therapie im heutigen zeitlichen Kontext. Ein Nutzen einer frühzeitigen Heparintherapie ist hier unklar. Eine retrospektive Analyse aus China zeigt unter diesen Einflüssen sogar, dass eine *innerklinische präinterventionelle* Therapie mit Heparin beim NSTE-ACS mit erhöhten Blutungsraten einhergeht, ohne dabei die Mortalität oder das Reinfarktrisiko günstig zu beeinflussen [30]. *Weder die aktuell verfügbaren Leitlinien noch publizierte Studiendaten begründen somit das häufig praktizierte präklinische Loading beim NSTE-ACS ohne Kriterien zur sofortigen invasiven Diagnostik*.

### Stärken und Limitationen

Die PRELOAD-Befragung unterliegt inhärenten Limitationen. Es konnten keine spezifische Zugangskontrolle oder Überprüfung der beruflichen Qualifikation erfolgen. Im Szenariendesign musste Komplexität gegen Lesbarkeit sowie Aufmerksamkeit abgewogen werden. Die Szenarien wurden daher explizit im Sinne der Fragestellung gestaltet, lassen aber differenzierte Rückschlüsse auf das individuelle Blutungs- und Ischämierisiko oder die Medikamentenadhärenz nicht zu. Die Fallvignetten wurden mit einer klaren Diagnose versehen. Alternative Ursachen des akuten Thoraxschmerzes wurden aus der Abwägungsentscheidung des Loadings somit als Variable zielgerichtet entfernt, dadurch wird die Übertragbarkeit in den Alltag eingeschränkt. Lokale Antikoagulationsstrategien wurden im Fragebogen nicht erfasst.

Die Studie kann bedingt durch das Design keine Kausalitäten aufzeigen, sie bleibt hypothesengenerierend. Für die Herzinfarktforschung im Bereich des NSTE-ACS sind aus den Studienbeobachtungen unter erfahrenen NÄ heraus folgende Fragen zukünftig zu adressieren:

- Gibt es einen Vorteil der prä- vs. innerklinischen Heparintherapie?
- Hat ein möglicher historischer Vorteil der Heparinvorbehandlung im Kontext einer heutzutage üblichen frühinvasiven Koronarangiographie (± 24 h) bei NSTEMI Bestand?
- Bietet bei vorbestehender oraler Antikoagulationsbehandlung die Heparintherapie einen prognostischen Nutzen?

## Fazit für die Praxis

- Präklinisches Loading ist bei beim ACS auch unabhängig von oraler Antikoagulation die bevorzugte Behandlungsstrategie.
- Antikoagulierte Patienten werden präferenziell mit einer ASS-Monotherapie behandelt.
- Beim STEMI bedeutet dies eine potenzielle Unterversorgung.
- Beim NSTE-ACS-Verdacht besteht eine präklinische Übertherapie.
- In Abwesenheit belastbarer Evidenz sollte bei Patienten ohne Hinweis auf eine Gefäßokklusion insbesondere das Heparin-Loading erst innerklinisch nach Bestätigung der Arbeitsdiagnose erfolgen.
- Loading sollte im Rahmen einer randomisierten kontrollierten Studie zum Schluss der Evidenzlücke beim NSTE-ACS auf den Prüfstand gestellt werden.

## Supplementary Information

Zusatzmaterial Fragebogen

Zusatzmaterial Tab. 1: Kriterien für eine sofortige invasive Strategie beim NSTE-ACS

Zusatzmaterial Tab. 2a: Einflussfaktoren der Loading-Entscheidung beim NSTE-ACS

Zusatzmaterial Tab. 2b: Einflussfaktoren der Loading-Entscheidung beim STEMI
