# Supplementary material for: Preclinical loading in patients with acute chest pain and acute coronary syndrome - PRELOAD survey
Source: Med Klin Intensivmed Notfmed. 2023 Nov 30;119(7):529–37. [Article in German] doi: 10.1007/s00063-023-01087-8 (PMC11461559; doi:10.1007/s00063-023-01087-8)
Supplement: Supplementary file 1 — Zusatzmaterial Fragebogen [file 63_2023_1087_MOESM1_ESM.pdf]

Teil A:

A1. Wie alt sind Sie (in Jahren)?

A2. Wie ist Ihr Geschlecht?

- Weiblich
- Männlich
- Divers

A3. Wie viele Jahre sind Sie bereits als Notärztin/Notarzt tätig (ganze Jahre)?

Im Fall von anteiligen Jahren: runden Sie bitte auf ab 6 Monaten pro angefangenes Jahr, runden Sie bitte ab bis zu 6 Montan pro angefangenes Jahr.

A4. Wie viele Stunden pro Monat arbeiten Sie durchschnittlich als Notärztin/Notarzt?

- < 24 Stunden
- 24-72 Stunden
- 72-120 Stunden
- > 120 Stunden

A5. Ihr überwiegendes Einsatzgebiet umfasst welche Stadt- und Bevölkerungsstruktur?

- Ländliche Region und Kleinstadt (<20.000 Einwohner)
- Mittelstadt (20.000 bis <100.000 Einwohner)
- Großstadt (ab 100.000 Einwohner)
- Metropole (ab 1.000.000 Einwohner)

A6. In welchem Fachgebiet führen Sie die Facharztbezeichung bzw. in welchem Fachgebiet erfolgt aktuell ihre Facharztweiterbildung?

- Innere Medizin und Kardiologie
- Innere Medizin ohne Kardiologie
- Chirurgie
- Anästhesie
- Andere Fachrichtung

A7. FALLBEISPIEL 1

<u>Präklinische Anamnese:</u>

61 jähriger männlicher Patient mit akutem Thoraxschmerz seit 2 Stunden, retrosternale Lokalisation mit Ausstrahlung in den linken Arm; keine Dauermedikation.

<u>Präklinische Befunde:</u>

Herzfrequenz: 60/min, Blutdruck 100/60mmHg, Atemfrequenz 21/min, SpO2: 92% unter Raumluft; ST-Streckenhebungen in den EKG-Ableitungen II, III, aVF und V6; reziproke ST-Senkungen in I, aVL und V1-3.

<u>Arbeitsdiagnose:</u>

STEMI der Hinterwand. Sie fahren das nächstgelegene Herzkatheterlabor mit 24h/7Tage Interventionsbereitschaft an, die Fahrtzeit beträgt 10-15 Minuten.

Wie gehen Sie in diesem Fall präklinisch mit „Loading“ (Plättchenhemmung, Antikoagulation) um?

- Sie applizieren einzig ASS i.v. (Standarddosis) ☐
- Sie applizieren einzig unfraktioniertes Heparin i.v. (Standarddosis) ☐
- Sie applizieren ASS i.v. und unfraktioniertes Heparin i.v. (jeweils Standarddosis) ☐
- Sie geben präklinisch keine Plättchenhemmung und kein Antikoagulanz. ☐

A8.   FALLBEISPIEL 2

Präklinische Anamnese:

52 jähriger männlicher Patient mit akutem Thoraxschmerz seit 4 Stunden; retrosternale Lokalisation, brennender Charakter und mit Ausstrahlung in den linken Arm, keine Dauermedikation.

Präklinische Befunde:

Herzfrequenz: 60/min, Blutdruck 140/80mmHg, Atemfrequenz 18/min, SpO2: 95% unter Raumluft; keine Erregungsleitungsstörungen, kein Schenkelblock, keine ST-Streckenveränderungen.

Arbeitsdiagnose:

Anginöser Thoraxschmerz, mögliches akutes Koronarsyndrom (NSTEMI, instabile Angina pectoris). Sie fahren die nächstgelegene Brustschmerzambulanz (Chest Pain Unit) an, die Fahrtzeit beträgt 10-15 Minuten.

Wie gehen Sie in diesem Fall präklinisch mit „Loading“ (Plättchenhemmung, Antikoagulation) um?

- Sie applizieren einzig ASS i.v. (Standarddosis) ☐
- Sie applizieren einzig unfraktioniertes Heparin i.v. (Standarddosis) ☐
- Sie applizieren ASS i.v. und unfraktioniertes Heparin i.v. (jeweils Standarddosis) ☐
- Sie geben präklinisch keine Plättchenhemmung und kein Antikoagulanz. ☐

A9. FALLBEISPIEL 3

<u>Präklinische Anamnese:</u>

61 jähriger männlicher Patient mit akutem Thoraxschmerz seit 2 Stunden, retrosternale Lokalisation mit Ausstrahlung in den linken Arm; paroxysmales Vorhofflimmern in der Anamnese, Dauermedikation: Rivaroxaban 20mg/Tag, Antihypertensivum, orales Antidiabetikum.

<u>Präklinische Befunde:</u>

Herzfrequenz: 60/min, Blutdruck 100/60mmHg, Atemfrequenz 21/min, SpO2: 92% unter Raumluft; ST-Streckenhebungen in den EKG Ableitungen V1-4; reziproke ST-Senkungen in in den EKG Ableitungen II, III, aVF.

<u>Arbeitsdiagnose:</u>

STEMI der Vorderwand. Sie fahren das nächstgelegene Herzkatheterlabor mit 24h/7Tage Interventionsbereitschaft an, die Fahrtzeit beträgt 10-15 Minuten.

Wie gehen Sie in diesem Fall präklinisch mit „Loading“ (Plättchenhemmung, Antikoagulation) um?

- Sie applizieren einzig ASS i.v. (Standarddosis) ☐
- Sie applizieren einzig unfraktioniertes Heparin i.v. (Standarddosis) ☐
- Sie applizieren ASS i.v. und unfraktioniertes Heparin i.v. (jeweils Standarddosis) ☐
- Sie geben präklinisch keine Plättchenhemmung und kein Antikoagulanz. ☐

A10. FALLBEISPIEL 4

Präklinische Anamnese:

52 jähriger männlicher Patient mit akutem Thoraxschmerz seit 4 Stunden; retrosternale Lokalisation, brennender Charakter und mit Ausstrahlung in den linken Arm; paroxysmales Vorhofflimmern in der Anamnese, Dauermedikation: Marcumar (Phenprocoumon) gesteuert nach INR-Wert (INR vom Vortag 2,5), Antihypertensivum, orales Antidiabetikum.

Präklinische Befunde:

Herzfrequenz: 60/min, Blutdruck 140/80mmHg, Atemfrequenz 18/min, SpO2: 95% unter Raumluft; keine Erregungsleitungsstörungen, kein Schenkelblock, keine ST-Streckenveränderungen

Arbeitsdiagnose:

Anginöser Thoraxschmerz, mögliches akutes Koronarsyndrom (NSTEMI, instabile Angina pectoris). Sie fahren die nächstgelegene Brustschmerzambulanz (Chest Pain Unit) an, die Fahrtzeit beträgt 10-15 Minuten.

Wie gehen Sie in diesem Fall präklinisch mit „Loading“ (Plättchenhemmung, Antikoagulation) um?

- Sie applizieren einzig ASS i.v. (Standarddosis) ☐
- Sie applizieren einzig unfraktioniertes Heparin i.v. (Standarddosis) ☐
- Sie applizieren ASS i.v. und unfraktioniertes Heparin i.v. (jeweils Standarddosis) ☐
- Sie geben präklinisch keine Plättchenhemmung und kein Antikoagulanz. ☐

A11. FALLBEISPIEL 5

<u>Präklinische Anamnese:</u>

61 jähriger männlicher Patient mit akutem Thoraxschmerz seit 2 Stunden, retrosternale Lokalisation mit Ausstrahlung in den linken Arm; paroxysmales Vorhofflimmern in der Anamnese, Dauermedikation: Marcumar (Phenprocoumon) gesteuert nach INR-Wert (INR vom Vortag 2,5), Antihypertensivum, orales Antidiabetikum

<u>Präklinische Befunde:</u>

Herzfrequenz: 60/min, Blutdruck 100/60mmHg, Atemfrequenz 21/min, SpO2: 92% unter Raumluft; ST-Streckenhebungen in den EKG Ableitungen V1-4; reziproke ST-Senkungen in in den EKG Ableitungen II, III, aVF.

<u>Arbeitsdiagnose:</u>

STEMI der Vorderwand. Sie fahren das nächstgelegene Herzkatheterlabor mit 24h/7Tage Interventionsbereitschaft an, die Fahrtzeit beträgt 10-15 Minuten.

<u>Wie gehen Sie in diesem Fall präklinisch mit „Loading“ (Plättchenhemmung, Antikoagulation) um?</u>

- Sie applizieren einzig ASS i.v. (Standarddosis) ☐
- Sie applizieren einzig unfraktioniertes Heparin i.v. (Standarddosis) ☐
- Sie applizieren ASS i.v. und unfraktioniertes Heparin i.v. (jeweils Standarddosis) ☐
- Sie geben präklinisch keine Plättchenhemmung und kein Antikoagulanz. ☐

A12. FALLBEISPIEL 6

<u>Präklinische Anamnese:</u>

52 jähriger männlicher Patient mit akutem Thoraxschmerz seit 4 Stunden; retrosternale Lokalisation, brennender Charakter und mit Ausstrahlung in den linken Arm; paroxysmales Vorhofflimmern in der Anamnese, Dauermedikation: Rivaroxaban 20mg/Tag, Antihypertensivum, orales Antidiabetikum

<u>Präklinische Befunde:</u>

Herzfrequenz: 60/min, Blutdruck 140/80mmHg, Atemfrequenz 18/min, SpO2: 95% unter Raumluft; keine Erregungsleitungsstörungen, kein Schenkelblock, keine ST-Streckenveränderungen

<u>Arbeitsdiagnose:</u>

Anginöser Thoraxschmerz, mögliches akutes Koronarsyndrom (NSTEMI, instabile Angina pectoris). Sie fahren die nächstgelegene Brustschmerzambulanz (Chest Paint Unit) an, die Fahrtzeit beträgt 10-15 Minuten.

Wie gehen Sie in diesem Fall präklinisch mit „Loading“ (Plättchenhemmung, Antikoagulation) um?

- Sie applizieren einzig ASS i.v. (Standarddosis) ☐
- Sie applizieren einzig unfraktioniertes Heparin i.v. (Standarddosis) ☐
- Sie applizieren ASS i.v. und unfraktioniertes Heparin i.v. (jeweils Standarddosis) ☐
- Sie verzichten auf ein Loading. Sie geben präklinisch keine Plättchenhemmung und kein Antikoagulanz. ☐

A13. FALLBEISPIEL 7

<u>Präklinische Anamnese:</u>

55 jähriger männlicher Patient mit beobachtetem Herzkreislaufstillstand, prompte Laienreanimation (Low-flow Zeit: 8 Minuten), unbekannte medizinische Vorgeschichte

<u>Präklinische Befunde:</u>

initialer Rhythmus Kammerflimmern, Rückkehr des Spontankreislaufs nach 2. Schockabgabe. Hämodynamisch stabiler Patient ohne Herzrhythmusstörungen und ohne Katecholaminbedarf. EKG unter Spontankreislauf ohne ST-Streckenveränderungen, kein Schenkelblock, ohne Erregungsleitungsstörungen

<u>Arbeitsdiagnose:</u>

Extraklinisches Reanimationsereignis mit einsetzendem Spontankreislauf und ohne ST-Streckenhebungen im 12-Kanal-EKG. Sie fahren das nächstgelegene Cardiac Arrest Center an, die Fahrtzeit beträgt 10-15 Minuten.

Wie gehen Sie in diesem Fall präklinisch mit „Loading“ (Plättchenhemmung, Antikoagulation) um?

- [ ] Sie applizieren einzig ASS i.v. (Standarddosis)
- [ ] Sie applizieren einzig unfraktioniertes Heparin i.v. (Standarddosis)
- [ ] Sie applizieren ASS i.v. und unfraktioniertes Heparin i.v. (jeweils Standarddosis)
- [ ] Sie verzichten auf ein Loading. Sie geben präklinisch keine Plättchenhemmung und kein Antikoagulanz.

A14. FALLBEISPIEL 8

Präklinische Anamnese:

55 jähriger männlicher Patient mit beobachtetem Herzkreislaufstillstand, prompte Laienreanimation (Low-flow Zeit: 8 Minuten), unbekannte medizinische Vorgeschichte. Vor dem Kollaps habe der Patient gemäß den umstehenden Personen über drückenden retrosternalen Brustschmerz geklagt.

Präklinische Befunde:

initialer Rhythmus Kammerflimmern, Rückkehr des Spontankreislaufs nach 2. Schockabgabe. Hämodynamisch stabiler Patient ohne Herzrhythmusstörungen und ohne Katecholaminbedarf. EKG unter Spontankreislauf ohne ST-Streckenveränderungen, kein Schenkelblock, ohne Erregungsleitungsstörungen

Arbeitsdiagnose:

Extraklinisches Reanimationsereignis mit einsetzendem Spontankreislauf und ohne ST-Streckenhebungen im 12-Kanal-EKG. Sie fahren das nächstgelegene Cardiac Arrest Center an, die Fahrtzeit beträgt 10-15 Minuten.

Wie gehen Sie in diesem Fall präklinisch mit „Loading“ (Plättchenhemmung, Antikoagulation) um?

- Sie applizieren einzig ASS i.v. (Standarddosis) ☐
- Sie applizieren einzig unfraktioniertes Heparin i.v. (Standarddosis) ☐
- Sie applizieren ASS i.v. und unfraktioniertes Heparin i.v. (jeweils Standarddosis) ☐
- Sie verzichten auf ein Loading. Sie geben präklinisch keine Plättchenhemmung und kein Antikoagulanz. ☐

A15. FALLBEISPIEL 9

Präklinische Anamnese:

52 jähriger männlicher Patient mit akutem, anhaltenden, drückenden Thoraxschmerz seit 4 Stunden; retrosternale Lokalisation, brennender Charakter und mit Ausstrahlung in den linken Arm; Dauermedikation: Antihypertensivum, orales Antidiabetikum

Präklinische Befunde:

Herzfrequenz: 92/min, Blutdruck 80/40mmHg, Atemfrequenz 25/min, SpO2: 90% unter Raumluft; nicht-anhaltende, selbstlimitierende ventrikuläre Tachykardie über eine Monitorlänge, ST-Streckensenkungen über der Vorderwand, keine ST-Streckenhebungen, kein Schenkelblock.

Arbeitsdiagnose:

Anginöser Thoraxschmerz, mögliches akutes Koronarsyndrom (NSTEMI, instabile Angina pectoris) mit kardiogenem Schock. Sie fahren die nächstgelegene Brustschmerzambulanz (Chest Paint Unit) an, die Fahrtzeit beträgt 10-15 Minuten.

Wie gehen Sie in diesem Fall präklinisch mit „Loading“ (Plättchenhemmung, Antikoagulation) um?

- Sie applizieren einzig ASS i.v. (Standarddosis) ☐
- Sie applizieren einzig unfraktioniertes Heparin i.v. (Standarddosis) ☐
- Sie applizieren ASS i.v. und unfraktioniertes Heparin i.v. (jeweils Standarddosis) ☐
- Sie verzichten auf ein Loading. Sie geben präklinisch keine Plättchenhemmung und kein Antikoagulanz. ☐

A16. Bestehen nach Ihrer Einschätzung explizite Leitlinienempfehlungen (nationale und europäische Ebene) zum Einsatz des präklinischen Loadings bei ST-Streckenhebungsinfarkt (STEMI)?

- Ja ☐
- Nein ☐
- Ich kenne die aktuellen Leitlinienempfehlungen nicht ausreichend zu Beurteilung der Frage. ☐

A17. Bestehen nach Ihrer Einschätzung explizite Leitlinienempfehlungen (nationale und europäische Ebene) zum Einsatz des präklinischen Loadings bei nicht-ST-Streckenhebungsinfarkt (NSTEMI)?

- Ja ☐
- Nein ☐
- Ich kenne die aktuellen Leitlinienempfehlungen nicht ausreichend zu Beurteilung der Frage. ☐

A18. Bestehen nach Ihrer Einschätzung explizite Leitlinienempfehlungen (nationale und europäische Ebene) zum Einsatz des präklinischen Loadings bei Patienten mit extraklinischer Reanimation ohne ST-Streckenhebungen nach Rückkehr des Spontankreislaufs?

- Ja ☐
- Nein ☐
- Ich kenne die aktuellen Leitlinienempfehlungen nicht ausreichend zu Beurteilung der Frage. ☐
