# Supplementary material for: Preclinical loading in patients with acute chest pain and acute coronary syndrome - PRELOAD survey
Source: Med Klin Intensivmed Notfmed. 2023 Nov 30;119(7):529–37. [Article in German] doi: 10.1007/s00063-023-01087-8 (PMC11461559; doi:10.1007/s00063-023-01087-8)
Supplement: Supplementary file 2 — Zusatzmaterial Tab. 1: Kriterien für eine sofortige invasive Strategie beim NSTE-ACS [file 63_2023_1087_MOESM2_ESM.docx]

| **Zusatzmaterial Tabelle 1: Kriterien für eine sofort invasive-Strategie beim NSTE-ACS** | |
| --- | --- |
| - | Kardiogener Schock oder hämodynamische Instabilität |
| - | Therapierefraktärere oder wiederholte Angina pectoris |
| - | Lebensbedrohliche Arrhythmien  (Herzkreislaufstillstand als Einzelfallentscheidung) |
| - | Akute Herzinsuffizienz als Infarktfolge |
| - | Mechanische Komplikation des Herzinfarktes |
| - | ST Segment Depression > 1mm/6 Ableitungen zzgl. ST-Streckenhebung in Ableitung aVR oder V1 |
| Modifiziert nach [Adler und Baldus 2020; Thiele et al. 2021 und Heyne et al. 2023]  Legende: NSTE-ACS: akutes Koronarsyndrom ohne ST-Streckenhebung | |
