# Supplementary material for: Preclinical loading in patients with acute chest pain and acute coronary syndrome - PRELOAD survey
Source: Med Klin Intensivmed Notfmed. 2023 Nov 30;119(7):529–37. [Article in German] doi: 10.1007/s00063-023-01087-8 (PMC11461559; doi:10.1007/s00063-023-01087-8)
Supplement: Supplementary file 3 — Zusatzmaterial Tab. 2a: Einflussfaktoren der Loading-Entscheidung beim NSTE-ACS [file 63_2023_1087_MOESM3_ESM.docx]

| **Zusatzmaterial Tabelle 2a): Einflussfaktoren der Loading Entscheidung NSTE-ACS**  **Chi-Quadrat Test und Regressionsanalyse** | | | | | | |
| --- | --- | --- | --- | --- | --- | --- |
|  | Alter | Geschlecht | Dienstjahre | Einsatzzeiten | Einsatzgebiet | Fachrichtung |
| **NSTE-ACS ohne Vorbehandlung** | 0,301  OR: 1,02 (95%CI: 1,001-1,047 ) | 0,349  # | 0,613  OR: 1,029 (95%CI: 1,003-1,056 ) | 0,11  OR: 2,63 (95%CI: 1,11-6,23 ) | 0,29  # | 0,05  # |
| **NSTE-ACS mit NOAK Vorbehandlung** | 0,186  # | 0,007  OR: 1,66 (95%CI: 1,18-2,33 ) | 0,04  # | 0,887  # | 0,667  # | 0,275  # |
| **NSTE-ACS mit Phenprocoumon Vorbehandlung** | 0,048  # | 0,008  OR: 1,67 (95%CI: 1,18-2,36 ) | 0,445  # | 0,666  # | 0,309  # | 0,075  # |
| **NSTE-ACS mit kardiogenem Schock** | 0,314  OR: 1,04 (95%CI: 1,007-1,073 ) | 0,367 | 0,833 | 0,247 | 0,119 | 0,795 |
| **Legende:** | * t-test einer Stichprobe, NSTE-ACS, NOAK, # kein valides Modell im Chi-Quadrat Likelihood Test | | | | | |
