# Supplementary material for: Preclinical loading in patients with acute chest pain and acute coronary syndrome - PRELOAD survey
Source: Med Klin Intensivmed Notfmed. 2023 Nov 30;119(7):529–37. [Article in German] doi: 10.1007/s00063-023-01087-8 (PMC11461559; doi:10.1007/s00063-023-01087-8)
Supplement: Supplementary file 4 — Zusatzmaterial Tab. 2b: Einflussfaktoren der Loading-Entscheidung beim STEMI [file 63_2023_1087_MOESM4_ESM.docx]

| **Zusatzmaterial Tabelle 2b): Einflussfaktoren der Loading Entscheidung STEMI**  **Chi-Quadrat Test und Regressionsanalyse** | | | | | | |
| --- | --- | --- | --- | --- | --- | --- |
|  | Alter | Geschlecht | Dienstjahre | Einsatzzeiten | Einsatzgebiet | Fachrichtung |
| **STEMI ohne Vorbehandlung** | - | - | - | - | - | - |
| **STEMI mit NOAK Vorbehandlung** | 0,886  # | 0,434  # | 0,975  # | 0,739  # | 0,008  * | 0,394  # |
| **STEMI mit Phenprocoumon Vorbehandlung** | 0,691  OR: 0,947 95% CI: 0,909-0,987 | 0,39  # | 0,775  # | 0,103  # | 0,115  # | 0,11  # |
| **Legende:** | STEMI; # kein valides Modell im Chi-Quadrat Likelihood Test | | | | | |
| ***** | OR  Großstadt vs. Ländliche Region 0,088 95% CI: 0,011-0,722  Großstadt vs. Mittelstadt 0,103 95% CI: 0,013-0,819  Großstadt vs. Metropole 0,472 95% CI: 0,029-7,611 | | | | | |
